# E-care 4 caregivers – an online intervention for nonprofessional caregivers of patients with depression: study protocol for a pilot randomized controlled trial

**DOI:** 10.1186/s13063-016-1320-6

**Published:** 2016-04-11

**Authors:** L. Bijker, A. Kleiboer, H. Riper, P. Cuijpers, T. Donker

**Affiliations:** Department of Clinical Psychology, Faculty of Movement and Behavioral Sciences, Vrije Universiteit Amsterdam, van der Boechorststraat 1, 1081 BT Amsterdam, the Netherlands; EMGO+ Institute for Health and Care Research, VU University Medical Center, Van der Boechorststraat 7, 1081 BT Amsterdam, the Netherlands

**Keywords:** E-mental health, Randomized controlled trial, Pilot study, Feasibility, Caregivers, Depression, Prevention, Burden, Psychological distress

## Abstract

**Background:**

Nonprofessional caregivers are highly important in the everyday life of patients with depression. Yet, they may experience increased levels of burden, stress, depression, and anxiety. Therefore, there is a need for interventions that relieve symptoms and are accessible and time-efficient. This paper describes the protocol of a pilot study to evaluate (1) the feasibility of an online self-management intervention, E-care 4 caregivers, for the nonprofessional caregiver of patients with depression, and (2) the initial effects of E-care 4 caregivers on psychological distress, subjective burden, symptoms of anxiety and depression, and quality of life.

**Methods/design:**

The study is a randomized controlled trial in which we are comparing the E-care 4 caregivers online intervention with a wait list control group. Eighty-four nonprofessional caregivers of patients with depression aged 18 years or older are being recruited from among the general population. Feasibility is determined by semistructured telephone interviews evaluating the subjects’ satisfaction with the intervention and by using a questionnaire on the user-friendliness of the system. The primary outcome measure used to examine the initial effects of the intervention is psychological distress. Secondary outcome measures are subjective burden, symptoms of anxiety and depression, level of mastery, and quality of life. Assessments will be done at baseline and 6 weeks later. Statistical analysis of the effects of the intervention will be carried out on the basis of the intention-to-treat principle.

**Discussion:**

E-care 4 caregivers could potentially benefit nonprofessional caregivers, as well as patients and professionals indirectly.

**Trial registration:**

Netherlands Trial Register identifier: NTR5268. Registered on 30 June 2015.

## Background

Nonprofessional caregivers are of high importance in the everyday lives of patients with depression. They may contribute significantly to day-to-day personal care and psychological support. The importance of nonprofessional caregivers has been recognized by the Dutch government, which encourages a greater role for caregivers in providing psychological support [1], as the care for these patients is virtually unattainable by professional health care providers alone. However, caring for a loved one with depression also has drawbacks, such as the risk of overburden due to decreased time for personal activities, disrupted everyday life [2], communication problems, financial difficulties, and social exclusion [3, 4]. This puts these nonprofessional caregivers at risk for developing symptoms of stress, anxiety, and depression, which often are associated with reduced quality of life [4–8]. Research by de Boer and colleagues [5] showed that 80 % of nonprofessional caregivers express a need for more support in the form of information, advice, or guidance.

Prevention research has demonstrated that early intervention is effective in preventing overburdening and decreasing distress. Stam and Cuijpers [9], for example, have demonstrated that a group psychoeducational intervention can reduce the experienced burden of nonprofessional caregivers of patients with a range of psychological problems. Likewise, a group psychoeducational intervention for nonprofessional caregivers caring for a patient with bipolar disorder, as compared with a control group, also demonstrated a reduction in experienced burden as well as an increase in knowledge about the disorder [10]. However, these group interventions are time-consuming and often do not fit into a caregiver’s busy schedule.

An online self-management intervention for the nonprofessional caregiver of a patient with depression that is aimed at increasing mental resilience and self-reliance to reduce subjective burden and prevent the development of psychological symptoms may overcome these barriers. It could be accessed 24 h per day, 7 days per week at a location and within a time frame that are the most convenient for the caregiver. Such an intervention would also be relatively easy to implement on a larger scale in the general population with minimal costs.

Previous research demonstrated robust evidence that Internet-based treatments can be effective for reducing symptoms of depression and anxiety [11, 12]. The effect sizes are comparable to those observed in face-to-face treatment [13]. However, Internet-based interventions have the potential to be more cost-effective than face-to-face treatment [14]. Also, an online intervention that was developed for nonprofessional caregivers caring for people with dementia was well-received (www.dementieonline.nl). One study showed positive results of web-based peer support for families of patients with depression, although the researchers in that study recommended including professional feedback as well [15]. However, to date, no study has examined the effects of online interventions based on cognitive behavioral therapy (CBT) principles for caregivers of patients with depression [16].

This paper describes the protocol of a pilot study designed to evaluate (1) the feasibility of an online self-management intervention, E-care 4 caregivers, for nonprofessional caregivers of patients with depression, and (2) the initial effects of E-care 4 caregivers on psychological distress, subjective burden, symptoms of anxiety and depression, level of mastery, and quality of life.

## Methods/design

This study is a two-arm randomized controlled trial (RCT) comparing the online intervention E-care 4 caregivers with a wait list control group (WLC). Assessments will be done at baseline and 6 weeks later.

### Procedure

**Fig. 1:**
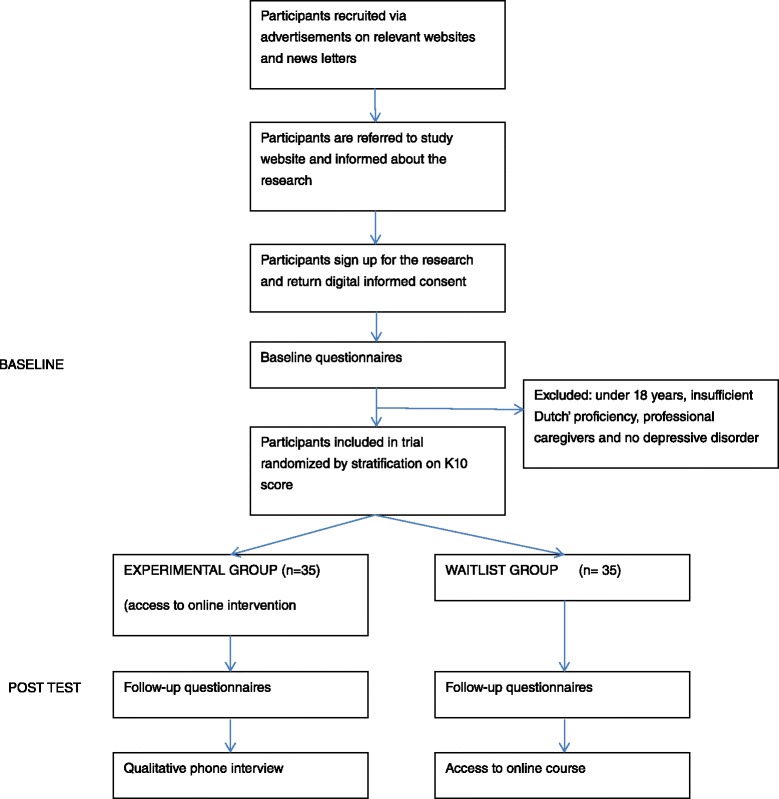
Flow diagram of the trial. *K10* Kessler Psychological Distress Scale

Nonprofessional caregivers will be recruited via the Dutch Depression Association and by advertisements on the Internet and other media (see Figure 1). Caregivers who are interested in taking part can find more information about the study on the website of the Mental Health Fund (Fonds Psychische Gezondheid), which hosts the study website. If they are interested in taking part in the study, they can register via the website. Next, they have to provide digital informed consent before being directed to the online baseline assessment. Those who are eligible to take part will be randomized to the intervention condition or the WLC group. Subjects in the intervention condition will have access to the online intervention for 6 weeks, while those assigned to the WLC condition will get access to the E-care 4 caregivers intervention after the 6-week assessment. Participants in both arms will be invited to complete an online assessment 6 weeks after baseline. Participants in the E-care 4 caregivers intervention group may also be approached for a qualitative interview. The Consolidated Standards of Reporting Trials (CONSORT) will be followed for reporting the trial [17]. Participants who complete all data entry points will receive an incentive of 25 euros.

### Study population

This study targets adult nonprofessional caregivers of loved ones with depression (partners, parents, children, siblings, family, or friends). Included are (self-reported) nonprofessional caregivers aged 18 years and older who care for a loved one with depression (including those with comorbid disorders). Excluded are nonprofessional caregivers without Internet access and those with no proficiency in Dutch.

#### Sample size

There are no definitive guidelines for sample sizes of pilot studies. On the basis of a simulation study [18], a sample size of 70 participants is recommended to estimate pooled standard deviations for a continuous variable. Taking into account an attrition rate of 20 %, we will include 84 respondents in total.

### Randomization and blinding

All participants eligible to take part will be randomized to the E-care 4 caregivers intervention or the WLC group, stratified by their scores on the K10 using a cutoff score of 20 [19]. The random allocation sequence and the randomization process will be performed by an independent researcher in the Random Allocation Software program. Due to the nature of the intervention, double-blinding of group allocation is not possible.

### Intervention: E-care 4 caregivers

The online self-management course comprises eight modules. The elements of it are described in Table 1. The modules include theory, exercises, examples of caregivers` experiences, and short videos. This intervention will include personalized feedback provided by a coach and a diary app. An Internet forum that is developed as a closed group on Facebook will also be a part of the online course. This forum is used for peer contact and is moderated by a coach.

**Table 1:** Content online intervention

Themes	Topics	Goals
1. What is depression?	Psychoeducation concerning symptoms, diagnosis, types, and causes of, as well as treatment options for, depression	To familiarize caregivers with the nature of the depressive disorder and its treatment to enable recognition of symptoms
2. Implications of depression for the caregiver	Emotions that can arise for the caregiver and the family, grief processes that occur, and practical implications of the depression	To recognize, validate, and accept negative emotions that can arise in a caregiver when facing the situation that a loved one has depression
3. How to think differently	Automatic thoughts, cognitive distortions, and how to correct these using principles of cognitive therapy	Using cognitive principles to recognize dysfunctional thoughts and change them to help caregivers in this stressful situation
4. Signaling stress and burnout	Psychoeducation about causes and signs of stress using the capabilities/workload model	Recognition and/or prevention of symptoms of overburdening and stress
5. Caring for yourself	Tips to better take care of yourself and your own boundaries with exercises about help-seeking, assertiveness, time-management, and relaxation exercises	Preventing and/or decreasing symptoms of overburdening and stress via various exercises
6. Learning to communicate better	Psychoeducation about communicating with someone with depression using the disarm technique and principles of emotionally focused therapy	To recognize your own role in communicating while understanding someone with depression better to avoid constant repetitive arguments
7. Coping with suicidality	Psychoeducation about suicidality, tips to communicate about suicidality, establishing and following safety procedures	Empowering caregiver with more knowledge about suicidality and safety measures as well as gaining more confidence in communication and setting boundaries
8. Caring for children of a parent with depression	Psychoeducation about possible consequences for the children of a parent with depression, with tips for communication, structure, and referral possibilities	Support parents to raise their children to become emotionally balanced individuals within a challenging environment where one parent has depression

#### Development of the intervention

The online self-management intervention is based on the self-help book *Depression: a guide for family members* [20]. This book is based on psychoeducation, CBT principles, and the experience of the book’s author in working with support groups for family members of depressed people.

Relevant stakeholders were also included in the early development phase of the intervention by means of two focus groups in which the needs of nonprofessional caregivers were assessed. The first focus group included five nonprofessional caregivers currently caring for a loved one with depression. The second focus group consisted of eight professionals in the field of depression, nonprofessional caregivers, and/or professionals in the design and use of Internet interventions.

#### Content

Both caregivers and experts agreed that the focus of the content should be on the caregiver, not on the patient with depression. Key components are recognition and validation for the nonprofessional caregiver. Psychoeducation about depression, stress, burnout, and self-care is needed, as well as practical information about general mental health care and tips regarding communication, grief, assertiveness, boundaries, and how to cope with negative feelings (guilt, responsibility, anger). Optional information for certain situations would be about coping with suicidality, changes in a relationship, and how to support children of a parent with depression.

#### Format

Both caregivers and experts preferred a cherry-picking design of the intervention (i.e., caregivers can choose which modules they would like to follow). Both groups agreed the content should be visually attractive and informal in tone, yet professional. Information should be provided by experts in an interactive manner by using text, videos, exercises, and examples. Interestingly, the caregivers differed in their recommendations about the frequency of use. Professionals preferred a more structured form, whereas nonprofessional caregivers recommended that the online course be available whenever they could find the time. Also, caregivers highly valued professional guidance by a coach, as they require validation and recognition of their stressful situation. The opportunity for peer contact would also be highly advisable.

#### Prerequisites

Both focus groups agreed that the online intervention should also be accessible via smartphones and tablets. Furthermore, it should be free of charge, password-protected, and run without technical difficulties. Also, it would be beneficial if participants could track their own progress to feel a sense of accomplishment.

### Assessments

All quantitative assessments are self-report measures and will be administered online. Semistructured qualitative interviews will be conducted by telephone. An overview of the questionnaires and their time of assessment is provided in Table 2.

**Table 2:** Overview of questionnaires and the time of assessment

Questionnaire	Baseline	6 weeks
Demographic variables, participant + depressed family member	X	
Kessler Psychological Distress Scale (K10)	X	X
Generalized Anxiety Disorder Scale (GAD7)	X	X
Zarit Burden Interview (ZBI-12)	X	X
Euro-Qol (EQ-5D)	X	X
System Usability Scale (SUS)		X
Pearlin Mastery Scale	X	X

### Feasibility instruments

The qualitative analysis to determine feasibility will be carried out using a structured telephone interview after subjects complete the online self-management course. In-depth interviews about what was experienced as valuable in the course, missing and redundant elements, and user-friendliness will be held with nonprofessional caregivers. Questions will also be asked regarding how well the course fits in with and contributes to their everyday lives and how they use the course to decrease their own burden. A topic list will be used that is based on the outcomes of the focus groups. The interview will start with the ‘grand tour question’: “What was your experience in using the online course?” The interviews will last between 45 minutes and 1 h and will be recorded for analysis.

#### User-friendliness

The user-friendliness of the online intervention is measured using the Dutch version of the System Usability Scale (SUS) [21]. The SUS is composed of 10 statements that are scored on a 5-point scale of strength of agreement. Final scores for the SUS can range from 0 to 100, where higher scores indicate better usability. This means that products that are at least passable have SUS scores above 70, with better products scoring in the high 70s to upper 80s. Truly superior products score better than 90. Products with scores less than 70 should be considered candidates for increased scrutiny and continued improvement and should be judged to be marginal at best. Reliability is good (Cronbach’s α 0.91).

### Initial effects instruments

#### Primary outcome measure

##### Psychological distress

Psychological stress will be measured by the translated ten-item version of the Kessler Psychological Distress Scale (K10) [22]. The K10 consists of ten items concerning feelings of depression, anxiety, and insecurity experienced in the last month. Subjects can score these feelings on a 5-point Likert scale (5 = “all of the time” to 1 = “none of the time”); the total score ranges from 10 (no distress) to 50 (severe distress). The reliability (Cronbach’s α) of the Dutch K10 was 0.94, and its validity was good (AUC 0.87). With a cutoff point of 20, the Dutch K10 reached a sensitivity of 0.80 and a specificity of 0.81 for any depressive and/or anxiety disorder [19].

#### Secondary outcome measures

##### Anxiety symptoms

Anxiety symptoms will be measured using the Dutch version of the 7-item Generalized Anxiety Disorder Scale (GAD7) [23]. The GAD7 consists of seven items concerning anxiety complaints experienced in the last 2 weeks. Subjects can score these feelings on a 4-point scale (0 = “not at all” to 3 = “nearly every day”); total score ranges from 0 to 21. With a cutoff point of 12, the web-based Dutch version is reliable and has a sensitivity of 0.83 and a specificity of 0.65 for generalized anxiety disorder [24].

##### Subjective burden

Subjective burden is measured with the Dutch version of the Zarit Burden Interview [25]. This questionnaire measures the consequences of long-term psychiatric or physical illnesses of patients on their nonprofessional caregivers. It consists of 12 items that are scored on a 5-point scale (0 = “never” to 4 = “almost all the time”); total score ranges from 0 to 48. Bedard and colleagues [26] found a Cronbach’s α greater than 0.88 for the overall scale and high values for the personal strain factor and the role strain factor (0.89 and 0.77, respectively).

##### Quality of life

Quality of life will be measured using the five-item self-report instrument EQ-5D [27]. The EQ-5D measures health-related quality of life and consists of five dimensions (mobility, self-care, main activity, pain, and mood). Each dimension is rated as causing “no problems,” “some problems,” or “extreme problems.” The EuroQol valuations appear to have good test-retest reliability. The EQ-5D thus distinguishes 486 unique health states. Each unique health state has a utility score that ranges from 0 (poor health) to 1 (perfect health). We used the single EQ-5D summary index score.

##### Perceived control

Perceived control of events and ongoing situations will be assessed using a five-item version of the Pearlin Mastery Scale [28] regarding how much control an individual perceives having over circumstances in his or her life. The scores per item vary from 1 (totally disagree) to 5 (totally agree). The Pearlin Mastery Scale has good psychometric properties and shows good reliability. Items are summed for a total mastery score (range from 5 to 25), with higher scores indicating greater perceived control (internal locus of control).

##### Other questions: general demographic characteristics

Information on general demographic variables such as age, sex, marital status, medication use, income, hours spent on caregiving, and educational level will be collected at baseline. Also, demographic variables related to the caregiver’s loved one are gathered, including age, sex, type of relationship, living situation, diagnosis, comorbidity, treatment, and suicidality.

### Analysis

#### Qualitative evaluation

Qualitative interview data will be analyzed using thematic content analysis [29]. The recorded interviews will be processed verbatim. Different themes will be identified and described using grounded theory principles [30]. The quality of the data collection will be guarded by a process of peer debriefing. Participants will be selected via purposive sampling on demographic variables (age, sex, type of relationship with the patient). Deviant cases will be actively searched for using information on the Internet forum or on the basis of SUS scores. Participants will be included until data saturation is reached.

#### Statistical analysis

Descriptive statistics will be used to report demographic variables, clinical outcomes, and the use of the different modules. The analysis of the quantitative primary and secondary measures will be performed on an intention-to-treat basis. To test the robustness of the findings, we will also conduct per-protocol analyses (those who completed the baseline and 6-week assessments and followed at least 50 % of the modules). Comparisons will be made between- and within groups for pre- and posttest measurements. For the intention-to-treat analyses for the continuous variables, repeated measures analysis of variance will be used. Standard effect sizes will be calculated (Cohen’s *d*) with confidence intervals. Sensitivity analyses will be performed on the primary outcome psychological distress (K10 score). IBM SPSS version 21 software (IBM, Armonk, NY, USA) will be used in analyses.

### Data monitoring and management

All study data will be captured electronically in a secured online survey platform (Qualtrics B.V., Amsterdam, the Netherlands; www.qualtrics.com). The data will be uploaded into the IBM SPSS database. All data will be kept in separate databases and merged into a master database only after data collection is completed and each individual database is locked. Qualitative data will be collected using audio recordings, written field notes, and memos. The data will be anonymized but linked with the trial identifier. The program Atlas.ti will be used to analyze the qualitative data. Recordings will be stored in a secure, password-protected folder. All data will be anonymized and electronically stored at the VU Amsterdam, separate from identifying information. Access to data will be password-protected. Only the lead investigators and trial researchers will have access to the final dataset. All collected data will be used only for the purposes of this research.

### Harms

The intervention is considered safe, with very low risk of adverse events. We estimate that the benefits of the study will be greater than any possible risk. Serious adverse events (e.g., negative life events, symptoms) are regularly monitored by the coach during the intervention. They will be documented by the research team and reported immediately to the principal investigator. If there is an adverse event, it will be discussed with a senior clinical academic independent of VU University and the research team. Appropriate courses of action will be agreed on and implemented. Participants are also advised to contact their general practitioner if they experience increased mental health symptoms.

### Ethical approval

This study is being conducted in accordance with local laws and regulations and follows the Medical Research Guidelines for Good Clinical Practice and European Union directives to ensure trial integrity as well as participants’ safety and well-being [31]. Respondents who are interested in taking part receive detailed information about the study and have the opportunity to ask questions. Digital informed consent will be obtained from all study subjects before the start of the study. Participants are allowed to terminate study participation at any time without giving a reason. The study has been reviewed and approved by the ethics committee of VU Amsterdam, Amsterdam, the Netherlands (VCWE-2015-126). Modifications to the protocol require a formal amendment to the protocol. This will be completed by the principal investigator. All changes and amendments to the protocol are supervised and approved by the sponsor.

## Discussion

To our knowledge, this study will be the first pilot RCT to evaluate the feasibility and initial effects of E-care 4 caregivers, an online self-management intervention for nonprofessional caregivers of patients with depression. Previous research among nonprofessional caregivers in general revealed an urgent need for support [5], and group interventions showed positive results in reducing stress and experienced burden [9, 10].

The strength of the study design is the inclusion of a qualitative analysis to assess the feasibility of the intervention. Further, this pilot study will provide a reliable indication of the sample size needed for an RCT testing the effectiveness of the intervention. Only preliminary conclusions on the effectiveness of the intervention can be drawn, owing to the small sample size. Conclusions regarding the prevention of disorders is, at this stage, preliminary. Also, this intervention targets the general population, and it is possible that potential improvement cannot yet be detected. However, the population is still at higher risk for psychological complaints [4–8], so a first indication would therefore be needed.

This intervention is one of the first [16] to be developed for this type of nonprofessional caregiver, and recruitment results as well as previous research underscore the need for more support in this particular group [5]. Also, the intervention has the potential to be cost-effective [14] and relatively easy to implement in general health care to reduce costs and increase availability for professional mental health care.

This study will provide insight into the feasibility and initial effects of an online self-management intervention for nonprofessional caregivers of patients with depression. E-care 4 caregivers could potentially benefit nonprofessional caregivers, as well as patients and professionals indirectly.

### Trial status

The trial started recruitment in September 2015.

## Abbreviations

CBT: cognitive behavioral therapy
CONSORT: Consolidated Standards of Reporting Trials
GAD7: 7-item Generalized Anxiety Disorder Scale
K10: Kessler Psychological Distress Scale
RCT: randomized controlled trial
SUS: System Usability Scale
WLC: wait list control group
ZBI-12: 12-item Zarit Burden Interview
